# An aid to interfacing analytical instrumentation to laboratory computer systems

**DOI:** 10.1155/S1463924687000099

**Published:** 1987

**Authors:** Ian Clark, Margaret Peters

**Affiliations:** Walfson Research Laboratories Queen Elizabeth Medical Centre Birmingham B15 2TH UK


**Journal of Automatic Chemistry**/*Journal of Clinical Laboratory Automation*, **Vol. 9, No. 1 (January–March 1987), pp. 44–45**

### An aid to interfacing analytical instrumentation to laboratory computer systems

**Ian Clark and Margaret Peters**

*Wolfson Research Laboratories, Queen Elizabeth Medical Centre, Birmingham B15 2TH, UK*

Most current laboratory computer systems are based on operating systems designed for multi-terminal access to a shared data-base, and not for real-time data acqusition from on-line analytical instruments. Several years ago this would have caused severe problems, because most instruments produced data output in the form of a varying analogue voltage which needed to be sampled, digitized, and monitored by a complex program which decided when a signal peak was reached. Such operations, particularly when required simultaneously for several instruments, are virtually impossible to achieve with a single computer at the same time as the multi-access data-base functions required for a modern laboratory system. This problem has been largely eliminated in recent years, because modern instruments produce data in digital form, normally using the RS232C standard employed for computer communications with printers, terminals etc. as outlined by DeBats and O'Meara [1]. Unfortunately, the use of the RS232C interface has introduced a new series of problems, and this paper outlines a low-cost method of eliminating some of these difficulties.

### Problems in interfacing

An RS232C interface transmits data as a serial stream of binary digits ('bits') along a pair of wires, one being used for data transmitted from a device, the other for data reception. Many other signal wires are included in the specification, most being intended for transmitting data over the telephone network via modems, but a few may be used with instrumentation (see below). The speed of data transfer (known as the 'baud rate', and measured in bits per second), can vary over a wide range, but in order to communicate, the devices at each end of an RS232C link must be set to the same rate. Data are encoded as 'characters', and the length of these (usually 7 or 8 bits) may vary. 'Stop' bits (1 or 2 bits) are appended to each character for use by the receiver circuitry and an additional 'parity' bit may be included in order to check correct transmission. All these parameters must match at each end of a link before transfers can take place. Computers and analytical instruments normally have facilities to enable any of the parameter settings to be altered so that a mutually compatible format is achieved. If this is not feasible, it may not be possible to establish a link.

A second problem is that of 'flow control'. Because the computer system and instrument are both required to perform several tasks 'simultaneously', it is probable that either one will, at certain times, be unable to receive data from the other because of higher priority demands. If data is not to be lost, some convention or protocol must be used by each device to signal when it is able to receive. There are several such protocols, some using signals on the additional control wires already mentioned, others using special control characters embedded in the data stream. Unfortunately few computers, and even fewer instruments offer a choice of flow controls: each manufacturer usually adopts a single standard, but not necessarily the same one.

A further difficulty related to flow control is the varying length of messages transmitted from instrument to computer system. Since, as already stated, most laboratory systems are built around operating systems designed for general data-base interrogation and update, data input is assumed to be via manually operated terminals, and the software is therefore designed to cope with only relatively short messages. If a compatible flow control protocol is not used, parts of long records are likely to be lost, since the computer will be unable to prevent transmission of data from the instrument whilst it processes previous segments of a long record.

The timing of signals between the two devices may also be important. Some instruments require reception of data to be acknowledged within a certain time. When a laboratory computer becomes overloaded for any reason, it may be unable to make the required response within the allowed time period (in which case it is said to have been 'timed-out'). 'No response' is generally interpreted by the instrument as indicating data lost or corrupted in transit, which often triggers a retransmission of the record just received.

Data transmitted between the instrument and computer system often contain a 'checksum'. This is a short message segment derived from the rest of message which is calculated by the transmitting device and added to the message, recalculated by the receiver, and compared with the transmitted value in order to check for corruption. Many different algorithms are used for such checksums and all are generally simple to calculate using the machine code employed within the instrument. This may be more difficult within a laboratory computer which uses a high level language not necessarily intended for this type of data manipulation. It is remotely possible that the language is incapable of calculating the checksum, but it is much more likely that calculation will be a slow process. We have encountered one configuration where the calculation time exceeded the instrument time-out period, so that successful reception could never be acknowledged. In this particular case, the problem was resolved simply by ignoring the checksum and forcing a retransmission of each record. This allowed the two copies to be compared in order to detect any corruption.

Most RS232C interfaces transmit characters using a coding convention known as ASCII (American Standard Code for Information Interchange). If a common coding system is not used at each end of the link, this incompatibility could be overcome by means of code translation within the software, but only at the expense of an increased system load. In addition, although ASCII purports to be a 'standard' coding system, some computers use slight variations and this may cause problems depending on the characters that are changed.

Finally, the successful physical interfacing of an analytical instrument and computer is only the first stage in the process of establishing on-line data acquisition. Another major consideration is the software required within the computer to accept the data and transfer this into the laboratory data-base. Any combination of these problems may occur, and in the past the commonest solution has been the use of a small personal computer as an 'intelligent' link between the two systems. The choice of such a machine is relatively limited, because it must either have two independent serial interfaces (a rare configuration), or some method of switching a single interface between the instrument and computer (even rarer). The costs may be high, ranging from several hundred to more than £1000, and, in addition, relatively complex software may be required in the linking machine in order to achieve a working system.

Further discussion of the above problems can be found in a paper by Broughton, Clark and Peters [2].

### A solution to some interfacing problems

In recent years, a small electronic device known as a 'printer buffer' has become available for use with desk-top office microcomputers. With these systems the computer often cannot be used for anything else whilst it is sending text to an associated printer. Consequently when large documents are being printed on low speed printers, the computer is unavailable for some time. Printer buffers have been developed to obviate this problem. The main components of such a device consist of a microprocessor, a large array of memory, two serial interfaces, each operating at an independent baud rate, and a read-only memory containing the progam which controls the function of the unit. The purpose of the circuit is to allow text to be sent from the computer into the memory array (or buffer) at the fastest possible rate, and then passed onwards to the printer at a rate it can accept. Provided there is sufficient memory to hold all, or most of a document, the computer then becomes available for other tasks before printing is complete. The cost of such devices usually ranges between £100 and £200.

Features of these devices which make them useful in interfacing analytical instrumentation are independent control of the baud rate on the two serial interfaces, and independently selectable character length, stop bits, parity and flow control protocols. Although one type of print buffer may not offer all protocols, it should be possible to find one to suit a particular combination of instrument and computer. These features should allow most RS232 incompatibilities to be easily resolved. In addition, since the device acts as a protocol converter, it serves to overcome problems of long data strings, so that the complete record can be transferred to the buffer, and then read into the computer in segments of appropriate size. In the same way, instruments capable of locally storing many results may overwhelm a computer system if a large batch is transmitted retrospectively. The sure of a buffer allows the computer to take in data at its own rate.

Timing, checksum and code conversion problems cannot normally be overcome with 'off-the-shelf' print buffers. However, as already mentioned, the function of these devices is totally controlled by the read-only memory within the unit. It is therefore possible that if the circuit could be re-programmed, then checksums and/or code conversion could be achieved within the print buffer. Although these units are usually marketed by large companies offering a wide range of data processing support products, they are often manufactured by small specialist electronics companies, which may be able to undertake customized reprogramming of the standard circuit. The cost of such a device will obviously be more than the basic unit, but should still be insignificant when compared with the combined cost of instrument and computer system. Alternatively, if the microprocessor and memory within the unit are standard devices, local reprogramming may be possible, if appropriate, where in-house expertise is available.

Recently we have developed a general clinical laboratory data processing system, based on DEC PDP-11 hardware and using MUMPS as a programming language. Baud rate, character length and parity are in the control of the programmer, but record length is limited to 255 characters, and a single flow control protocol is available. Print buffers have been successfully used to interface PDP-11 computers to a Technicon SMA2, Technicon H1, Cobas Fara and a Hitachi 737.

Where a print buffer is used as described, i.e. as a baud rate, data format and protocol converter between instrument and computer system, consideration must be given to the long-term reliability of the unit. Since these devices are so inexpensive that maintenance contracts are inappropriate, the purchase of a spare would seem to be indicated.

### Acknowledgements

The authors ackowledge financial support by the Department of Health and Social Security.

### References

1. De Bats, A. and O'Meara, J. (Eds), *A Guide to Data Processing in Clinical Laboratories* (Association of Clinical Biochemists, London, 1983).
2, Broughton, P. M. G., Clark, I. R. and Peters, Margaret, *Annals of Clinical Biochemistry*, **18** (1986), 585.

